# Stress-Induced Changes in the Lipid Microenvironment of β-(1,3)-d-Glucan Synthase Cause Clinically Important Echinocandin Resistance in Aspergillus fumigatus

**DOI:** 10.1128/mBio.00779-19

**Published:** 2019-06-04

**Authors:** Shruthi Satish, Cristina Jiménez-Ortigosa, Yanan Zhao, Min Hee Lee, Enriko Dolgov, Thomas Krüger, Steven Park, David W. Denning, Olaf Kniemeyer, Axel A. Brakhage, David S. Perlin

**Affiliations:** a Center for Discovery and Innovation, Hackensack Meridian Health, Nutley, New Jersey, USA; b Public Health Research Institute, New Jersey Medical School, Rutgers Biomedical and Health Sciences, Newark, New Jersey, USA; c Leibniz Institute for Natural Product Research and Infection Biology (HKI), Jena, Germany; d National Aspergillosis Centre, Wythenshawe Hospital, University of Manchester, Manchester Academic Health Science Centre, Manchester, United Kingdom; e Department of Microbiology and Molecular Biology, Institute of Microbiology, Friedrich Schiller University, Jena, Germany; University of Toronto

**Keywords:** *Aspergillus fumigatus*, ROS, antifungal resistance, caspofungin, echinocandins, glucan synthase, glucan synthase inhibitors, lipids

## Abstract

Aspergillus fumigatus is a leading cause of invasive fungal infections. Resistance to first-line triazole antifungals has led to therapy with echinocandin drugs. Recently, we identified several high-minimum-effective-concentration (MEC) A. fumigatus clinical isolates from patients failing echinocandin therapy. Echinocandin resistance is known to arise from amino acid substitutions in β-(1,3)-d-glucan synthase encoded by the *fks1* gene. Yet these clinical isolates did not contain mutations in *fks1*, indicating an undefined resistance mechanism. To explore this new mechanism, we used a laboratory-derived strain, RG101, with a nearly identical caspofungin (CAS) susceptibility phenotype that also does not contain *fks1* mutations. Glucan synthase isolated from RG101 was fully sensitive to echinocandins. Yet exposure of RG101 to CAS during growth yielded a modified enzyme that was drug insensitive (4 log orders) in kinetic inhibition assays, and this insensitivity was also observed for enzymes isolated from clinical isolates. To understand this alteration, we analyzed whole-enzyme posttranslational modifications (PTMs) but found none linked to resistance. However, analysis of the lipid microenvironment of the enzyme with resistance induced by CAS revealed a prominent increase in the abundances of dihydrosphingosine (DhSph) and phytosphingosine (PhSph). Exogenous addition of DhSph and PhSph to the sensitive enzyme recapitulated the drug insensitivity of the CAS-derived enzyme. Further analysis demonstrated that CAS induces mitochondrion-derived reactive oxygen species (ROS) and that dampening ROS formation by antimycin A or thiourea eliminated drug-induced resistance. We conclude that CAS induces cellular stress, promoting formation of ROS and triggering an alteration in the composition of plasma membrane lipids surrounding glucan synthase, rendering it insensitive to echinocandins.

## Abstract (precis)

Resistance to first-line triazole antifungal agents among *Aspergillus* species has prompted the use of second-line therapy with echinocandins. As the number of *Aspergillus*-infected patients treated with echinocandins is rising, clinical observations of drug resistance are also increasing, indicating an emerging global health threat. Our knowledge regarding the development of clinical echinocandin resistance is largely derived from *Candida* spp., while little is known about resistance in *Aspergillus.* Therefore, it is important to understand the specific cellular responses raised by A. fumigatus against echinocandins. We discovered a new mechanism of resistance in A. fumigatus that is independent of the well-characterized *FKS* mutation mechanism observed in *Candida*. This study identified an off-target effect of CAS, i.e., ROS production, and integrated oxidative stress and sphingolipid alterations into a novel mechanism of resistance. This stress-induced response has implications for drug resistance and/or tolerance mechanisms in other fungal pathogens.

## INTRODUCTION

Invasive fungal infections are a serious threat to human health, causing at least 1.5 million deaths worldwide each year (1). Such infections are common in immunocompromised patients and are associated with high rates of mortality: 30% to 40% for invasive candidiasis and 30% to 90% for invasive aspergillosis (2, 3). Patients with invasive or chronic pulmonary aspergillosis are typically treated with azoles as a first-line therapy. However, resistance to azoles is on the rise and second-line therapy with echinocandins is therefore becoming prominent (4).

Mechanisms of echinocandin resistance have been extensively studied in yeasts of the *Candida* genus. In these organisms, clinical resistance to echinocandins arises via mutations in the hot spot regions of *FKS* genes which encode the cell wall biosynthetic enzyme β-(1,3)-d-glucan synthase (5). While *fks1* mutations have also been linked to resistance to echinocandins in Aspergillus fumigatus (6, 7), high-minimum-effective-concentration (MEC) echinocandin-resistant clinical strains of A. fumigatus containing a wild-type (WT) copy of *fks1* have also been identified (8). In addition, it was reported that upregulation of glucan synthase may also result in reduced clinical drug response (9). These observations point to the clinical relevance of *fks1* mutation-independent mechanisms for echinocandin resistance in A. fumigatus.

β-(1,3)-d-Glucan synthase catalyzes the addition of glucose monomers to the growing chain of β-(1,3)-d-glucan (10, 11). It is predicted to be an integral membrane protein with 16 transmembrane domains, a putative cytoplasmic N terminus, and a putative UDP-glucose binding sequence at the C terminus (12–16). Yet echinocandin binding and inhibition are not well defined. It has been speculated that the lipid-like tail of echinocandins may intercalate into the hot spot regions of glucan synthase, resulting in inhibition (17). In Candida glabrata, mutants in certain lipid biogenesis pathways showed differential susceptibilities to different echinocandins, a finding that was hypothesized to be due to changes in lipid composition near the enzyme (18). However, how the modulation of glucan synthase results in altered echinocandin sensitivity has not been examined.

RG101 is a spontaneous high-MEC A. fumigatus mutant derived from ATCC 13073 generated in Perlin laboratory. This strain is resistant to caspofungin (CAS) but contains no mutations in the *fks1* gene (19). Therefore, to begin to examine *fks1-*independent mechanisms of echinocandin resistance in *Aspergillus*, we investigated the mechanism of RG101 resistance to CAS and then extended our findings to the echinocandin-resistant clinical isolates of A. fumigatus. Our study identified a novel stress-induced mechanism of echinocandin resistance in *Aspergillus* that is mediated by mitochondrion-derived reactive oxygen species (ROS). This clinically important mechanism induces drug insensitivity of glucan synthase by modulating its immediate lipid environment. It reflects an important adaptation response in fungal species.

## RESULTS

### A. fumigatus echinocandin resistance independent of *fks1* mutations.

Clinical isolates of A. fumigatus obtained from patients with chronic pulmonary aspergillosis who failed echinocandin therapy were shown to have elevated MECs for both CAS and micafungin (MFG) (Table 1). DNA sequence analysis revealed no mutations in the *fks1* gene open reading frame or promoter (data not shown), suggesting that the mechanism of echinocandin resistance in these strains was independent of the established *FKS* mechanism of well-characterized *Candida* species (4) and known to exist in Aspergillus fumigatus (6, 7). The level of *fks1* expression was not increased upon CAS induction (see Fig. S1 in the supplemental material), indicating that overexpression of the drug target was not the mechanism of resistance in RG101.

**TABLE 1:** Minimum effective concentrations of clinical isolates of A. fumigatus from patients with chronic pulmonary aspergillosis who failed echinocandin therapy

Aspergillus fumigatus clinical isolate	Isolate source	MEC^*a*^		Nature of *fks1* gene
		CAS (μg/ml)	MFG (μg/ml)	
32458	Sputum	16	2	WT
32315	Sputum	16	2	WT
117	Sputum	16	4	WT
2770	Sputum	16	2	WT
68284B	Sputum	4	4	WT
ATCC 13073	Human pulmonary lesion	0.12	0.03	WT
S679P	Laboratory-generated echinocandin-resistant mutant	16	16	Ser-679-Pro

a MEC, minimum effective concentration.

10.1128/mBio.00779-19.2 FIG S1 *fks1* expression levels in RG101 under uninduced and CAS-induced conditions. RG101 conidia were grown for 16 h in YPD in the absence and presence of CAS (1 and 4 μg/ml), and expression levels of *fks1* were compared using reverse transcription-PCR (RT-PCR). No significant differences in *fks1* expression levels were seen under uninduced and CAS-induced conditions (*P* > 0.05). Download FIG S1, DOCX file, 0.02 MB. Copyright © 2019 Satish et al. 2019 Satish et al. This content is distributed under the terms of the Creative Commons Attribution 4.0 International license.

**FIG 1:**
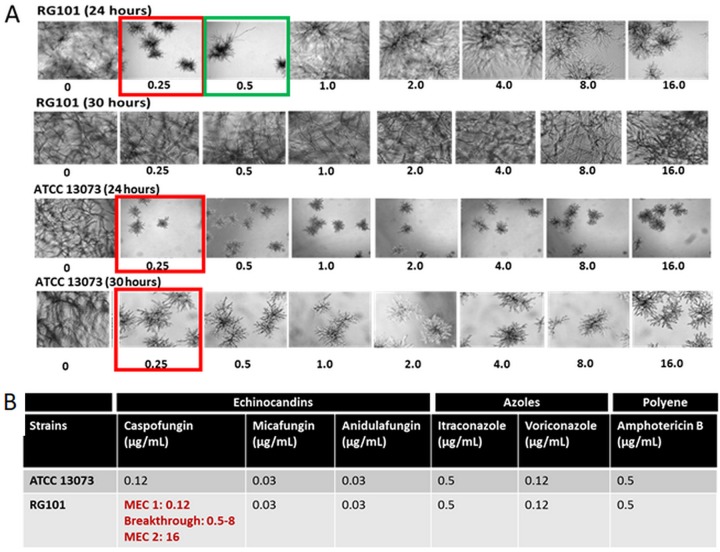
RG101 shows breakthrough growth in CAS. (A) Time-dependent changes in growth phenotypes of RG101 and ATCC 13073 in RPMI 1640 medium. At 24 h, the MEC of CAS for RG101 was 0.25 μg/ml, with the formation of characteristic rosettes indicating inhibition (red). However, breakthrough growth began to manifest at 0.5 μg/ml (green), and at between 1 and 8 μg/ml of CAS, this strain showed complete resistance. At 16 μg/ml, rosettes began to form again, indicative of growth inhibition. At 30 h, resistance of RG101 to CAS was seen at all concentrations of CAS tested. (B) Results of drug susceptibility testing of RG101 and ATCC 13073 at 24 h in the presence of different antifungals. RG101 was resistant only to CAS but was sensitive to other echinocandins, azoles, and polyenes. ATCC 13073 was sensitive to all antifungals.

To better understand this potential novel resistance mechanism, we turned our attention to a previously reported laboratory-generated strain of A. fumigatus called RG101, which exhibited a drug susceptibility phenotype comparable to those seen with the echinocandin-resistant and *fks1* wild-type (WT) clinical isolates. The RG101 strain was spontaneously generated following CAS exposure of echinocandin-susceptible parental strain ATCC 13073 (19). The resulting mutant strain displayed an unusual paradoxical high-resistance phenotype but was devoid of any mutation in the *fks1* gene. At 24 h, RG101 was sensitive to CAS with an MEC of 0.25 μg/ml, with the formation of characteristic rosette structures indicating growth inhibition. However, breakthrough growth began to manifest at 0.5 μg/ml, and at 1 and 8 μg/ml of CAS, this strain showed complete resistance. At 16 μg/ml, rosettes began to form again, indicative of drug sensitivity (Fig. 1A). By 30 h, full breakthrough was seen at all concentrations of CAS tested (0.25 to 8 μg/ml) (Fig. 1A). This phenotype, showing partial inhibition at low drug levels followed by full breakthrough growth at higher levels, suggested that there was drug-mediated induction of caspofungin resistance. As reported previously (19), RG101 was resistant to CAS and sensitive to all other antifungals, indicative of CAS-specific, inducible resistance phenotype (Fig. 1B).

**FIG 2:**
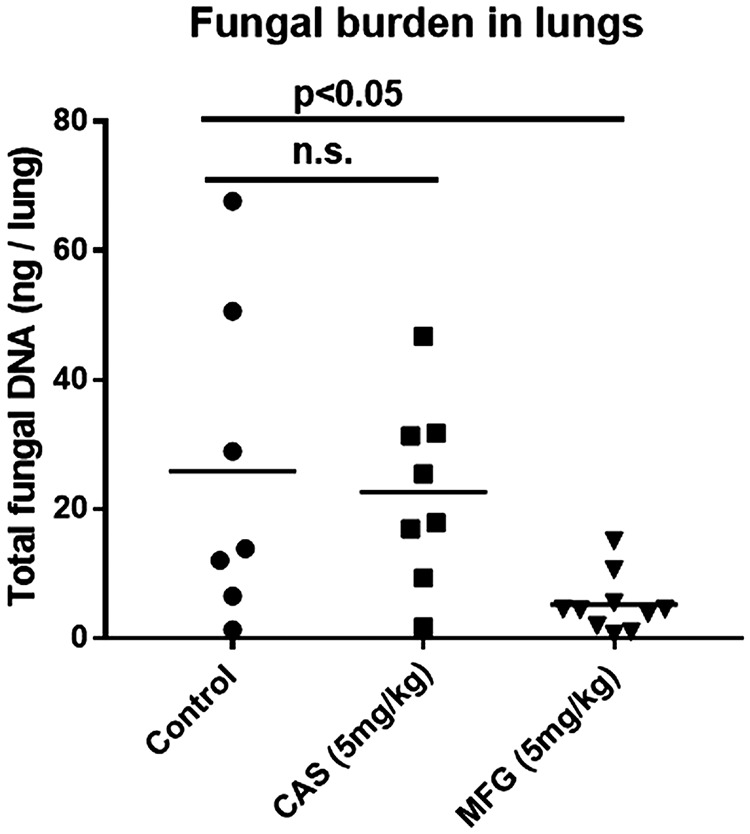
RG101 is resistant to caspofungin and sensitive to micafungin *in vivo*. Resistance of RG101 to CAS *in vivo* was tested in an acute pulmonary aspergillosis model. Inbred DBA/2 mice were rendered neutropenic and infected with 1 × 10^6^ RG101 conidia intratracheally. The mice were then treated with CAS, micafungin, or vehicle daily for 5 days, and lung burdens were determined using qPCR. The average fungal lung burdens in animals treated with vehicle, CAS, and MFG were 25.8, 22.6, and 5.2 ng fungal DNA per lung, respectively. Statistical analysis showed no significant differences between the vehicle-treated and CAS-treated groups, whereas MFG-treated animals showed approximately 5-fold decrease in burden, suggesting that RG101 is resistant to CAS and sensitive to MFG *in vivo*.

To determine if RG101 was resistant to CAS *in vivo*, we tested the strain in a murine model of pulmonary invasive aspergillosis, in which neutropenic DBA/2 mice were infected with RG101 conidia. The lung burdens of mice treated with CAS, MFG, or vehicle are presented in Fig. 2. Animals treated with vehicle, CAS, and MFG showed 25.8, 22.6, and 5.2 ng fungal DNA per lung, respectively. No significant differences in lung burden were observed between the vehicle-treated and CAS-treated groups, whereas the MFG-treated animals showed an approximate 5-fold decrease in burden, suggesting that RG101 was resistant to CAS but sensitive to MFG *in vivo*.

### Probing the *fks1*-independent resistance mechanism of RG101.

Echinocandin activity is known to induce compensatory changes in cell wall composition, resulting in cell wall alterations that can partially protect the fungus from the drug (20). We therefore examined potential ultrastructural changes in the cell wall upon CAS exposure using electron microscopy (EM) to obtain insights into the mechanism of resistance. In both scanning electron microscopy (SEM) and transmission electron microscopy (TEM) studies, RG101 grown in the presence of CAS did not show any ultrastructural cell wall damage. In contrast, the echinocandin-sensitive parent, ATCC 13073, showed extensive damage, irregular cell wall shape, and leaking of cellular contents (Fig. S2). We then examined the possibility that compensatory upregulation of other cell wall components, such as chitin, was driving the resistance in RG101. Biochemical analysis of cell wall composition by mass spectrometry (MS) and chitin content evaluation by calcofluor white staining showed no significant increase of chitin levels in RG101 upon CAS exposure (Fig. S3). We concluded that a compensatory increase in the level of chitin in the cell wall was not a likely mechanism of echinocandin resistance in RG101.

10.1128/mBio.00779-19.3 FIG S2 Cell wall ultrastructure of RG101 in the presence of CAS. Conidia of ATCC 13073 and RG101 strains were grown in liquid YPD in the presence of CAS (4 μg/ml) for 16 h and processed for SEM and TEM imaging. Electron microscopy images showed no morphological defects in RG101 grown in the presence of CAS, whereas the CAS-sensitive parental strain ATCC 13073 showed irregular mycelia and leaking cellular contents indicating damaged cell wall. Download FIG S2, DOCX file, 0.9 MB. Copyright © 2019 Satish et al. 2019 Satish et al. This content is distributed under the terms of the Creative Commons Attribution 4.0 International license.

10.1128/mBio.00779-19.4 FIG S3 Evaluation of glucan and chitin contents of RG101 cell wall (A) Biochemical analysis of cell wall glucan levels using mass spectroscopy showing no reduction in glucan content in RG101 treated with CAS. (B and C) Fluorescence study using calcofluor white showing no increase in chitin levels in RG101 treated with CAS (4 μg/ml). In summary, no cell wall-associated changes were detected in RG101 treated with CAS, indicating complete resistance to the drug. Download FIG S3, DOCX file, 0.4 MB. Copyright © 2019 Satish et al. 2019 Satish et al. This content is distributed under the terms of the Creative Commons Attribution 4.0 International license.

### The *fks1*-independent mechanism of resistance is mediated by CAS-induced modification of glucan synthase.

**FIG 3:**
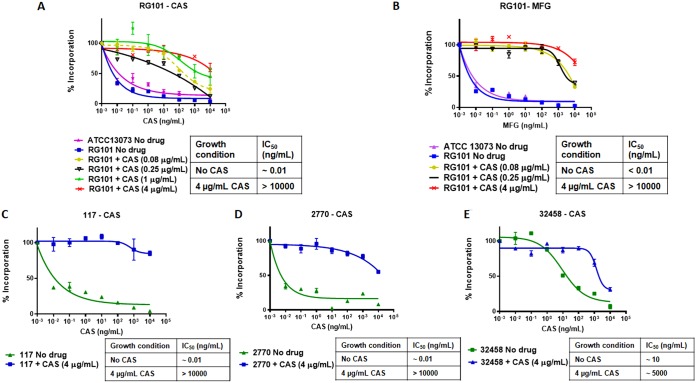
CAS induces modification of β-glucan synthase in RG101 and several clinical isolates. Sensitivity of partially purified glucan synthase to different echinocandins was examined *in vitro* after isolating the enzyme from RG101 grown in different drug conditions. (A) Dose-dependent insensitivity of RG101 glucan synthase to CAS induced by CAS during mycelial growth of RG101. The IC_50_ of glucan synthase for RG101 grown in the absence of CAS [IC_50_ (no CAS)] was ∼0.01 ng/ml and for RG101 grown at 4 μg/ml CAS [IC_50_ (4 μg/ml CAS)] was high at >10,000 ng/ml. Previous exposure to CAS in RG101 culture induced insensitivity to CAS at the enzyme level in a dose-dependent fashion. (B) Previous exposure to CAS in culture induced insensitivity to micafungin at the enzyme level [IC_50_ (no CAS), <0.01 ng/ml; IC_50_ (4 μg/ml CAS), >10,000 ng/ml], indicating potential CAS-induced cross-resistance in cells. (C to E) Enzyme profiles similar to those seen with CAS were observed in clinical isolates 117 (C), 2770 (D), and 32458 (E). IC_50_ values for the three clinical isolates are as follows: for isolate 117, IC_50_ (no CAS) = ∼0.01 ng/ml and IC_50_ (4 μg/ml CAS) = >10,000 ng/ml; for isolate 2770, IC_50_ (no CAS) = ∼0.01 ng/ml and IC_50_ (4 μg/ml CAS) = >10,000 ng/ml; for isolate 32458, IC_50_ (no CAS) = ∼10 ng/ml and IC_50_ (4 μg/ml CAS) = ∼5,000 ng/ml.

To further examine whether glucan synthase of RG101 was intrinsically resistant to inhibition by CAS, RG101 and corresponding parental strain ATCC 13073 were grown in the absence and presence of resistance-inducing levels of CAS. The active enzyme was partially purified, and the sensitivity of the enzyme to echinocandins was analyzed in kinetic inhibition assays (21). We observed that glucan synthase isolated from RG101 cultured in the absence of CAS was fully sensitive to inhibition by CAS or MFG (Fig. 3A and B). However, when glucan synthase was isolated from RG101 grown in the presence of CAS, it was 4 to 5 log orders less sensitive (as determined by the enzyme’s higher 50% inhibitory concentration [IC_50_] value) than that of RG101 grown in the absence of the drug (Fig. 3A). This biochemical insensitivity was not observed with the WT parental strain ATCC 13073. The drug-mediated reduced sensitivity correlated with increasing effector concentrations of drug in the growth media; i.e., RG101 cells grown in the presence of higher concentrations of CAS had higher IC_50_ values and thus were more resistant to CAS inhibition (Fig. 3A). Strikingly, glucan synthase isolated from RG101 cultured in the presence of CAS was also insensitive to MFG (Fig. 3B). These results strongly suggested that culturing RG101 in the presence of a CAS-induced modification(s) in glucan synthase had rendered it largely insensitive to all echinocandin class drugs.

To assess whether clinical isolates (Table 1) would show similar enzymatic behavior upon CAS induction, cells were cultured in the absence and presence of CAS (4 μg/ml), and the glucan synthase enzymes were isolated and evaluated in the kinetic inhibition assay. Indeed, three clinical isolates—117, 2770, and 32458—yielded enzymes with CAS-induced drug insensitivity, similar to RG101 (Fig. 3C and D and E), indicating that modification of glucan synthase induced by CAS is a conserved mechanism of echinocandin resistance in A. fumigatus.

### CAS induces cross-resistance to other glucan synthase inhibitors in RG101.

**FIG 4:**
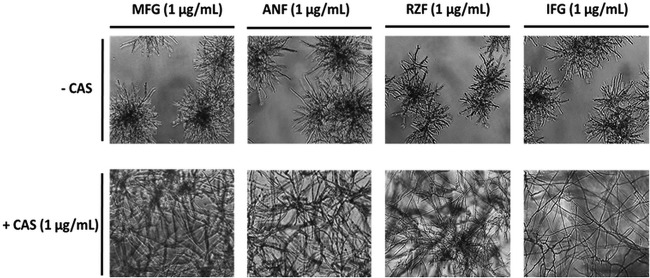
CAS induces cross-resistance to other glucan synthase inhibitors in RG101. RG101 was grown for 24 h in RPMI 1640 media containing 1 μg/ml of different glucan synthase inhibitors, including micafungin (MFG), anidulafungin (ANF), rezafungin (RZF), and ibrexafungerp (IFG), in both the absence and presence of CAS (1 μg/ml). While RG101 was susceptible to these drugs in the absence of CAS, as seen by characteristic formation of rosettes, it showed complete resistance in the presence of CAS, as seen by the presence of extended mycelia.

Since glucan synthase of RG101 grown in the presence of CAS showed enzyme insensitivity not only to CAS but also to MFG, we examined whether cross-resistance was also observed in liquid growth culture. To this end, we performed drug susceptibility testing of ATCC 13073 and RG101 with different glucan synthase inhibitors individually and in combination with an inducing level (1 μg/ml) of CAS. RG101 was sensitive to micafungin (MFG), anidulafungin (ANF), rezafungin (RZF), and ibrexafungerp (IFG) when grown in the presence of each drug alone. However, when these drugs were present in combination with 1 μg/ml of CAS, cross-resistance was observed (Fig. 4). This suggested that CAS induces cross-resistance to other glucan synthase inhibitors in RG101, paralleling the kinetic inhibition studies.

### Nature of glucan synthase modification leading to resistance in RG101.

We hypothesized that CAS induces resistance in RG101 by direct modification of glucan synthase through posttranslational modifications (PTMs). To test this hypothesis, glucan synthase was isolated from RG101 grown in the absence and presence of CAS (1 μg/ml) and subjected to enzymatic digestion and analysis by a nano-LC-MS/MS (liquid chromatography-tandem mass spectrometry) method to identify any potential PTMs. The peptides covered a significant portion (up to 71.5%) of the enzyme, including the major hot spot regions known to confer resistance. This analysis resulted in three important findings (Fig. S4). First, the A. fumigatus glucan synthase enzyme was decorated with numerous PTMs—methylation, acetylation, and phosphorylation—throughout the length of the protein. Second, no PTMs were detected in the hot spot regions of the enzyme. Third, a subset of peptides had PTMs in the induced enzyme sample that were absent in the uninduced enzyme. However, only a very small fraction (<5%) of the enzyme population was modified, making it unlikely to account for the observed phenotype. On the basis of these data, we believed it improbable that any PTM present at such low abundance would be responsible for the resistance phenotype (Fig. S4).

10.1128/mBio.00779-19.5 FIG S4 PTMs identified in glucan synthase. Nano-LC/MS analysis of glucan synthase of RG101 after enzyme digestion covered 71.5% of the protein and detected several PTMs. (A) A list of different PTMs detected with specific amino acid locations. (B) A diagrammatic representation of glucan synthase 16 transmembrane domains and the overall distribution of PTMs detected. Most of the PTMs were studded in the cytosolic segments of the protein. No PTMs were detected in the two hot spot regions of the enzyme. (C) List of 8 peptides showing differences in PTMs between CAS-induced and uninduced enzymes. However, a very low fraction of the population of the enzyme was modified, which did not account for the observed phenotype in RG101. Download FIG S4, DOCX file, 0.2 MB. Copyright © 2019 Satish et al. 2019 Satish et al. This content is distributed under the terms of the Creative Commons Attribution 4.0 International license.

**FIG 5:**
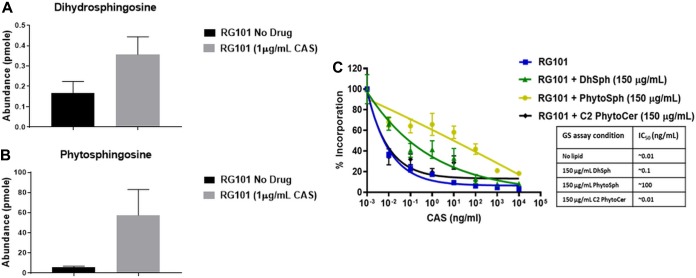
Certain lipids affect the sensitivity of β-glucan synthase to CAS. (A and B) Lipidomics analysis of enriched glucan synthase from RG101 grown in the absence and presence (1 μg/ml) of CAS showed (A) >2-fold-increased abundance of dihydrosphingosine (DhSph) and (B) 10-fold-increased abundance of phytosphingosine (PhytoSph) in CAS-exposed cells. (C) Addition of DhSph and PhytoSph at a concentration of 150 μg/ml for 1 h to enriched glucan synthase extract in the enzyme assay made the enzyme insensitive to CAS [IC_50_ (no lipid) = ∼0.01 ng/ml; IC_50_ (150 μg/ml DhSph) = 0.1 ng/ml; IC_50_ (150 μg/ml PhytoSph) = ∼100 ng/ml], whereas addition of other lipids such as phytoceramide did not alter the enzyme property [IC_50_ (150 μg/ml C2 PhytoCer) = ∼0.01 ng/ml].

It was previously reported for Candida glabrata that a change in sphingolipid content could alter whole-cell susceptibility to CAS and MFG (18). Thus, a comprehensive lipidomics analysis was performed to compare lipid profiles of glucan synthase containing fractions derived from RG101 grown in the absence and presence of CAS (see Table S1 in the supplemental material). In samples derived from CAS-treated cells, two lipid molecules—dihydrosphingosine (DhSph) and phytosphingosine (PhSph)—were found in increased abundance (2-fold and 10-fold, respectively) compared to untreated cells (Fig. 5A and B). These lipid species also showed significantly increased abundance in enzyme preparations of clinical isolates (117, 2770, and 32458) treated with CAS (Fig. S5).

10.1128/mBio.00779-19.6 FIG S5 Abundance levels of DhSph and PhSph lipid species in clinical isolates: Glucan synthase of three clinical isolates listed in Table 1—2770, 117, and 32458—was tested for abundance levels of DhSph and PhSph under uninduced and induced conditions. Consistent with data from RG101, these clinical isolates also showed higher levels of DhSph (A) and PhSph (B) in CAS-induced enzymes than in preparations of uninduced enzymes. This indicated that CAS induces lipid modifications in clinical isolates, as observed in RG101. Download FIG S5, DOCX file, 0.04 MB. Copyright © 2019 Satish et al. 2019 Satish et al. This content is distributed under the terms of the Creative Commons Attribution 4.0 International license.

10.1128/mBio.00779-19.8 TABLE S1 Relative abundances of different lipid species in the microenvironment of glucan synthase. Three lipid subtypes (in red)—dihydrospingosine (DhSph), phytosphingosine (PhSph), and phytoceramide (C24)—were present in CAS-induced glucan synthase of RG101 at levels 3-, 10-, and 3-fold higher, respectively, than those seen with the uninduced preparation. DhSph and PhSph were used for further analysis. Download Table S1, DOCX file, 0.02 MB. Copyright © 2019 Satish et al. 2019 Satish et al. This content is distributed under the terms of the Creative Commons Attribution 4.0 International license.

To further test whether DhSph and PhSph contribute to the insensitivity of the enzyme to CAS, these lipids were added exogenously to the kinetic inhibition assay reaction mixture containing the partially purified glucan synthase in detergent. Interestingly, each lipid dramatically altered the sensitivity profile of glucan synthase derived from RG101 grown in the absence of CAS. DhSph and PhSph increased the CAS IC_50_ value of the sensitive enzyme by at least 1 and 4 log orders, respectively (Fig. 5C). These effects were lipid dose dependent (data not shown). The observed effects were specific to DhSph and PhSph, as other lipid intermediates such as phytoceramide (C20) did not change the enzymatic sensitivity (Fig. 5C). This indicated that the composition of the lipid microenvironment surrounding the glucan synthase most likely altered its conformation in a way that is important for CAS enzyme inhibition.

### CAS induces production of reactive oxygen species in A. fumigatus.

**FIG 6:**
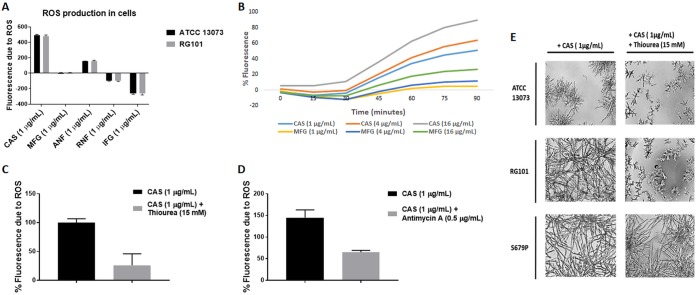
CAS induces ROS production. (A) Levels of ROS induced by different echinocandins as measured by a fluorescence assay using DCFDA dye. After 1 h of exposure of cells to echinocandins, CAS induced the highest levels of ROS production in the A. fumigatus RG101 and ATCC 13073 strains in comparison to other echinocandins. (B) Dose-dependent increase in ROS production induced by CAS. (C) Addition of thiourea, a ROS scavenger, at 15 mM reduced CAS-induced ROS production in RG101. (D) Addition of antimycin A, a mitochondrial respiration inhibitor, at 0.5 μg/ml reduced CAS-induced ROS production in RG101, indicating mitochondrion-associated ROS production induced by CAS. (E) Testing of susceptibility of ATCC 13073, RG101, and S679P strains with CAS (1 μg/ml) and thiourea (15 mM) revealed reversion of the resistance phenotype of RG101 but no change of the phenotype of ATCC 13073 and S679P.

As described previously, the induction of resistance in RG101 was specific to CAS (Fig. 1), and the cells showed cross-resistance to other echinocandins in the presence of CAS. Thus, we investigated several potential factors that might differentiate CAS from other echinocandins and observed that only CAS strongly induced the production of reactive oxygen species (ROS) in cells (Fig. 6). A 2′,7′-dichlorofluorescin diacetate (DCFDA)-based fluorescence assay was used to measure ROS levels in A. fumigatus cells. After 1 h of drug exposure, CAS induced the highest levels of ROS in cells compared to other glucan synthase inhibitors (Fig. 6A). The levels of ROS induced by the drugs were very similar between the ATCC 13073 and RG101 strains. There was a dose-dependent increase in ROS production induced by CAS (Fig. 6B). Consistent with the induction of ROS, RNA-seq analysis of RG101 harvested in the presence and absence of CAS induction revealed that the oxidation-reduction pathway was one of the most highly differentially expressed pathways, consistent with the production of ROS induced by CAS (Fig. S6). Next, the effect of thiourea, a ROS scavenger, on ROS levels and CAS resistance was tested. As expected, treatment with 15 mM thiourea reduced the levels of ROS induced by CAS by 4-fold (Fig. 6B). Addition of the mitochondrial respiration inhibitor antimycin A (0.5 μg/ml) to the assay decreased ROS production induced by CAS by over 2-fold, indicating that CAS-induced ROS is generated in mitochondria (Fig. 6C). Importantly, thiourea reversed the CAS-induced breakthrough resistance phenotype of RG101 but had no effect on CAS resistance in the *fks1-*S679P strain (Fig. 6D). These results suggest that CAS indirectly induces the production of mitochondrial ROS, which in turn leads to downstream modification of glucan synthase and echinocandin resistance via changes in the lipid microenvironment.

10.1128/mBio.00779-19.7 FIG S6 Differentially expressed genes and gene ontology data from comparisons performed under CAS-induced and uninduced conditions in RG101. RG101 was grown in the absence and presence of CAS (1 μg/ml) for 16 h, and cells were isolated and processed for RNA-seq. Genes involved in the oxidation-reduction pathway were highly differentially expressed, consistent with the induction of ROS by CAS. Download FIG S6, DOCX file, 0.6 MB. Copyright © 2019 Satish et al. 2019 Satish et al. This content is distributed under the terms of the Creative Commons Attribution 4.0 International license.

## DISCUSSION

In this study, a novel mechanism of resistance to echinocandins in Aspergillus fumigatus was investigated. Although azoles represent the primary treatment option for *Aspergillus* infections, therapeutic use of echinocandins is rising rapidly due to increasing emergence of azole resistance (22). The echinocandin caspofungin was first approved by the FDA in 2002 for use against *Aspergillus* infections refractory to conventional antifungal therapy (23). As the number of patients with *Aspergillus* infections being treated with echinocandins has expanded, reports of clinical failure of echinocandin therapy are emerging (6, 9). In *Candida* species, echinocandin resistance is tightly associated with *FKS* gene mutations that result in amino acid substitutions in hot spot regions of glucan synthase enzyme (21, 24–26). These mutations decrease the sensitivity of the enzyme to echinocandins by up to 3,000-fold (21, 25, 26). Additionally, certain cell wall stress adaptation response pathways become activated upon echinocandin exposure, resulting in elevated MIC values *in vitro* (27). However, these alterations are typically associated with drug tolerance and not with clinical failure. Drug tolerance represents an intermediate stage in the evolution of clinical resistance involving acquisition of characteristic *FKS* gene mutations. Tolerance mechanisms depend on fungal stress tolerance pathways, including cell wall integrity, protein kinase C, Ca^2+^- calcineurin/Crz1, and high-osmolarity glycerol (HOG) pathways (5). In one study, a systems biology approach was employed to infer regulatory networks and interactions during adaptation to CAS-induced stress in A. fumigatus (28). They confirmed the presence of cross talk of mitogen-activated protein (MAP) kinases corresponding to cell wall integrity and HOG signaling pathways upon CAS exposure. Data also suggested that intracellular transport was affected upon CAS exposure causing additional osmotic stress but that high concentrations of CAS reduced this osmotic stress, thus decreasing its toxicity. In this report, we describe a novel, stress-induced mechanism of echinocandin resistance in *Aspergillus* that is associated with clinical failure but independent of genetic changes leading to amino acid substitutions in the hot spot region of glucan synthase. This novel mechanism is mediated by an increase in ROS production induced prominently by CAS, resulting in a downstream modification of the lipid microenvironment, which likely alters the interaction of echinocandin drugs with glucan synthase.

### Alternative mechanism of echinocandin resistance.

Echinocandin resistance in *Aspergillus* is a relatively new phenomenon that involves modulation of the drug-target interaction mediated via two separate mechanisms of action (MOA). The first MOA is highly conserved and occurs through mutations in the hot spot regions of glucan synthase. It has been shown that an engineered S679P amino acid substitution in glucan synthase in a laboratory-generated A. fumigatus strain rendered it highly resistant to all echinocandins (7). More recently, our laboratory reported an echinocandin-resistant clinical isolate of A. fumigatus carrying the point mutation F675S in the hot spot region of the *fks1* gene (6). For the second MOA, echinocandin resistance in A. fumigatus has also been reported for high-MEC isolates from patients failing therapy in which there was no apparent genetic modification of *fks1* (9). In this case, upregulation of *fks1* was presumed to be a causal mechanism of resistance, comparable to what has been observed for gain-of-function mutations resulting in azole resistance in Candida albicans (29). In that report, clinical isolates from patients with chronic pulmonary aspergillosis who failed echinocandin therapy were analyzed and found to have elevated MECs with no mutations in the coding or noncoding regions of *fks1*. Furthermore, the strains did not show significant upregulation of *fks1*, pointing to a novel resistance mechanism. Previously, it was reported that a highly drug-resistant laboratory-generated strain, RG101, showed high-MEC CAS values with no apparent modification of the *fks1* gene (19). That strain displayed paradoxical drug sensitivity in which growth was inhibited at low levels of CAS but showed frank resistance at drug levels above 0.5 μg/ml after 24 h. That phenotype was recapitulated in the present study, and RG101 was shown to be fully resistant at all drug levels after 30 h of growth. The strain also appeared to be resistant *in vivo*. However, it retained complete sensitivity to MFG at 24 and 48 h and *in vivo*. Transcriptional analysis of RG101 grown in the presence of CAS at breakthrough levels of drug revealed that the cells upregulated a variety of stress and tolerance pathways associated with cell wall biosynthesis and remodeling, as had been observed previously in Candida albicans (19). However, these compensatory responses were insufficient to account for prominent drug resistance. This conclusion is consistent with a study by Loiko and Wagener, who analyzed the paradoxical behavior of *Aspergillus* under conditions of CAS treatment and suggested that the paradoxical drug recovery reflected a restoration of enzyme function (30).

To further explore a novel mechanism directly impacting glucan synthase, we examined the enzyme isolated from untreated and CAS-treated RG101 cells to explore a potential modification(s) of the drug target. Indeed, we observed that the glucan synthase from RG101 grown in the presence of CAS was altered, rendering the enzyme insensitive to the drug (4-log order shift in sensitivity). Furthermore, the modified enzyme was subsequently found to be insensitive to MFG and other glucan synthase inhibitors. Consistently, CAS induced cross-resistance to all glucan synthase inhibitors in whole-cell growth inhibition assays. This indicated that CAS was inducing some form of enzyme modification that rendered cells resistant. The possibility of PTMs in the enzyme causing insensitivity to CAS was tested using LC-MS/MS. Several PTMs were detected in glucan synthase of RG101, but no modification was judged to be stable and strong enough to drive such a steady resistance phenotype. The role of the observed PTMs in enzyme function and/or regulation is being independently evaluated.

Further, potential lipid-associated modifications in the microenvironment of the enzyme were examined, and we found two types of lipids—dihydrosphingosine and phytosphingosine—that were highly abundant in product-entrapped glucan synthase after CAS induction compared to the results seen with an uninduced-enzyme preparation. Exogenous addition of these lipids to the sensitive glucan synthase of RG101 was found to have made the enzyme insensitive to CAS. It is noteworthy that the clinical isolates with resistance phenotypes showed a similar lipid profile. This suggests that changes in the lipid bilayer can alter the interaction of echinocandins with glucan synthase. The role of the lipid microenvironment in the susceptibility of fungi to echinocandins has not been studied extensively. However, a study showed that for some laboratory and clinical isolates of C. glabrata, defects in sphingolipid biosynthesis led to a mixed phenotype in which strains were resistant to CAS and hypersensitive to MFG (18, 31). The authors of that study hypothesized that changes in sphingolipid composition in the cell membrane weakened the interaction between glucan synthase and CAS and strengthened the interaction between the enzyme and MFG. A recent study of C. albicans showed that deletion mutants of genes encoding enzymes important for biosynthesis of very-long-chain fatty acids such as sphingolipids resulted in resistance to fluconazole, an azole antifungal agent targeting the ergosterol biosynthesis pathway (32). Our findings indicate that CAS-induced changes in sphingolipid composition in the microenvironment of glucan synthase alter the drug-binding affinity of echinocandins. Therefore, this apparent nexus of two drug classes with very different MOAs—azoles and echinocandins—suggests that sphingolipid biosynthesis may play an important role in fungal cell stress tolerance responses.

### Oxidative stress response as a mediator of echinocandin resistance.

Studies have shown that the polyene drug amphotericin B (AmB) kills fungal cells by forming membrane pores but also acts as an oxidizing agent (33, 34), leading to the formation of ROS (35). The efficacy of the drug has been partially attributed to oxidative damage in cells through lipid peroxidation (36). Recently, a study in A. fumigatus showed that fungal cell membrane-targeting drugs such as itraconazole, terbinafine, and AmB induce mitochondrial ROS production, leading to lipid peroxidation and inhibition of fungal growth (37). Although all echinocandins share the same mechanism of action involving inhibition of glucan synthase, it is well documented that CAS induces *in vitro* phenotypic behavior (leading to high MICs) that is independent of genetic changes in *FKS*. For example, certain *Candida* strains grown in the presence of CAS show various degrees of paradoxical growth in which isolates are able to grow better at higher drug concentrations (38, 39). This effect is partially attributed to compensatory chitin deposition (40, 41), although this model is largely insufficient for *Aspergillus* (30). Overall, the biological mechanism behind these observations is not well understood. In this study, we investigated why CAS but not other echinocandins was able to induce modification of glucan synthase from RG101. We observed that CAS prominently induced ROS production, while the other echinocandins induced ROS weakly or not at all. The CAS-induced ROS was of mitochondrial origin, as it was blocked by antimycin A. Importantly, we showed a link between ROS production and development of resistance, as addition of ROS scavengers such as thiourea reduced ROS levels in cells and reversed the resistance phenotype of RG101. A few studies have linked increased ROS content to protein and lipid oxidation in cells (42). In one study in Saccharomyces cerevisiae, mitochondrial-respiration-deficient mutants were hypersensitive to CAS (42, 43). The most sensitive mutants were those with impaired mitochondrial biosynthesis of the phospholipids phosphatidylethanolamine and cardiolipin (43–46). Another study in S. cerevisiae showed that the TORC2/Ypk1 pathway functions as a ROS-sensing module and plays a central role in sphingolipid biosynthesis and homeostasis (47). How ROS production, induced by CAS, leads to altered lipid composition with respect to glucan synthase resulting in resistance is yet to be determined.

### A new model for stress-induced drug resistance.

**FIG 7:**
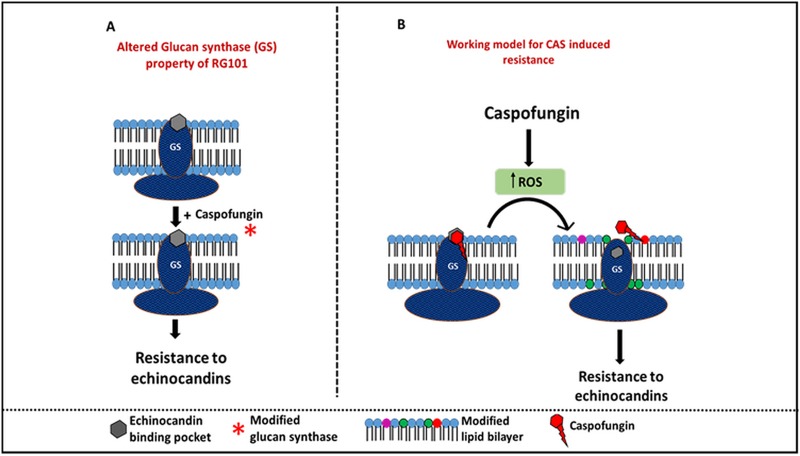
Working model showing the non-*fks1* mutation-mediated mechanism of resistance in RG101. (A) Addition of CAS during growth of RG101 altered the properties of glucan synthase, rendering it resistant to CAS, at both the cellular and enzyme levels. (B) Working model for CAS-induced resistance in RG101. CAS induces ROS production in cells. We hypothesize that high ROS levels alter the lipid composition in the microenvironment of glucan synthase, causing a conformational change in glucan synthase and leading to CAS resistance.

Our model for non-*fks1* mutation-mediated echinocandin resistance in A. fumigatus is shown in Fig. 7. We propose that CAS induces production of ROS in mitochondria as an off-target effect. The other echinocandins showed poor inductive effect, most likely due to poor cell penetration. We believe that one of the downstream effects of increased ROS production in cells is altered regulation of lipid biosynthesis genes. This leads in turn to modified plasma membrane lipid composition in the microenvironment of glucan synthase, likely causing a conformational change in the enzyme, perhaps in a manner that prevents echinocandins from binding to the target and leads to resistance. Although our data demonstrate the CAS-specific insensitivity of the enzyme upon addition of dihydrosphingosine and phytosphingosine, we hypothesize that insensitivity to all echinocandins may occur with much higher concentrations of these modified lipids. Clinical isolates have phenotypes that highly similar but not identical to those seen with RG101, since they are also MFG resistant. However, these isolates were retrieved from patients with chronic pulmonary aspergillosis who had undergone prolonged treatment with MFG and other antifungal therapy. Such a chronic exposure to drugs could have triggered similar pathways such as ROS production leading to altered lipid composition. Alternatively, prolonged exposure to micafungin may have lowered the threshold for activation of sphingolipid biosynthesis pathway genes in these strains, leading to altered lipid composition in the microenvironment of the enzyme.

In conclusion, this study identified a novel, *fks1-*independent mechanism of echinocandin resistance in A. fumigatus that uses CAS-induced ROS-mediated changes in the lipid composition of the microenvironment of glucan synthase to alter the drug-target interaction. Other echinocandins may also induce off-target mitochondrial stress (although less robustly than CAS), resulting in lower ROS production. This is yet to be assessed with large collections of clinical isolates. Use of echinocandins to treat patients with *Aspergillus* infection is expanding, and we predict that this new mechanism of resistance will be an important clinical factor.

## MATERIALS AND METHODS

### Strains and isolates.

RG101 was derived from parental strain ATCC 13073 (U.S. clinical isolate collected from a lung in 2005) by regenerating spheroplasts in minimal medium containing a high (10 μg/ml) concentration of CAS (19). A. fumigatus clinical isolates 117, 2770, 32458, 32315, and 68284B were obtained from sputum samples of patients with chronic pulmonary aspergillosis at Wythenshawe Hospital, University of Manchester (the samples were provided by David W. Denning). Strain S679P (previously reported as strain S678P) (7) was engineered in the Perlin laboratory to harbor an amino acid substitution in the hot spot region (amino acid 679) making it resistant to all echinocandins.

### Antifungal susceptibility testing.

Minimum effective concentrations (MECs) for A. fumigatus strains were determined according to CLSI document M38-A2 (48) and defined as the lowest drug concentration that led to the growth of small, rounded, compact hyphal forms compared to the hyphal growth seen in the drug-free growth control wells. The following drugs (supplier) were tested: caspofungin (Merck & Co., Inc.), micafungin (Astellas Pharma US, Inc.), anidulafungin (Pfizer Inc.), itraconazole (Sigma-Aldrich), voriconazole (Pfizer Inc.), and amphotericin B (Sigma-Aldrich). In addition, rezafungin and ibrexafungerp were obtained from Cidara Therapeutics and Scynexis Inc., respectively.

### *In vivo* fungal burden analysis.

Resistance of RG101 to CAS *in vivo* was tested in an acute pulmonary aspergillosis model. Thirty inbred DBA/2 mice were rendered neutropenic and infected with 1 × 10^6^ RG101 conidia intratracheally. The mice were then treated with CAS (5 mg/kg of body weight), MFG (5 mg/kg), or vehicle daily for 5 days, and lung burdens were determined using quantitative PCR (qPCR). Details of the experiment are provided in Text S1 in the supplemental material.

10.1128/mBio.00779-19.1 TEXT S1 Supplemental Materials and Methods: *in vivo* fungal burden analysis, protein analysis by LC-MS/MS, and electron microscopy. Download Text S1, DOCX file, 0.03 MB. Copyright © 2019 Satish et al. 2019 Satish et al. This content is distributed under the terms of the Creative Commons Attribution 4.0 International license.

### Electron microscopy.

Scanning electron microscopy (SEM) and transmission electron microscopy (TEM) methods were followed as described previously (49) and in Text S1.

### Cell wall carbohydrate analysis.

Strains ATCC 13073 and RG101 were grown in the absence and presence of CAS (4 μg/ml) in 6-well microtiter plates containing yeast extract-peptone-dextrose (YPD) broth for 22 h at 37°C. The cells were then lysed using a French press. The broken cell wall sample was processed for chemical analysis using mass spectrometry at the Complex Carbohydrate Research Center (CCRC) at the University of Georgia, Athens, GA.

### Glucan synthase isolation and assay.

ATCC 13073 or RG101 was grown in liquid YPD medium at 37°C for 16 h. Partially purified glucan synthase was isolated by the product entrapment method, as previously described (21). Sensitivity to echinocandin drugs was measured by glucan synthase assay, using a 96-well multiscreen high-throughput screen filtration system (Millipore Corporation, Bedford, MA) in a final volume of 100 μl. Inhibition of the enzyme by the drug was analyzed after incubation of the enzyme with a radioactive substrate over a broad range of concentrations (0 to 10,000 ng/ml) of echinocandins for 1 h at room temperature. Radioactivity from the enzyme product, β-(1,3)-d-glucan, was measured. Inhibition profiles were determined using a sigmoidal response curve fitting algorithm with GraphPad Prism software (v 7.04).

To study the effect of lipids on the enzyme, dihydrosphingosine, phytosphingosine, or phytoceramide C2 (Sigma-Aldrich D6783, P2795, and C8105, respectively) was added directly to the assay mixture and coincubated with an echinocandin for 1 h at room temperature.

### Protein analysis by LC-MS/MS.

To detect possible posttranslational modifications in glucan synthase, we assayed the enzyme from strain RG101 grown in the absence or presence (1 μg/ml) of CAS. To remove detergent from partially purified glucan synthase, the enzyme sample was spun at 39,000 rpm for 1 h in an ultracentrifuge. The pellets were resuspended in enzyme buffer. Protein samples were separated on SDS-PAGE, and bands of >180 kDa were collected and prepared for mass spectrometry **(**Text S1).

### Lipid analysis.

Approximately 10 μg/ml of partially purified glucan synthase of RG101 and clinical isolates was analyzed for lipid content by LC-MS/MS (50) at the Lipidomics Shared Resource of the Medical University of South Carolina (http://www.hollingscancercenter.org/research/shared-resources/lipidomics/index.html).

### Measurement of ROS production.

ROS production in mycelia was measured using a protocol previously described by Blatzer et al. (51) with a few changes. Suspensions of 10^4^ spores/ml of ATCC 13073 and RG101 were grown in static culture in complete supplement mixture (CSM) medium for 16 h in a 96-well microplate at 37°C. After incubation, the mycelia were washed once with CSM, 10 μM 2′,7′–dichlorofluorescin diacetate (DCFDA) dye was added, and cells were incubated in the dark at 37°C for 30 min. Cells were then washed three times with phosphate-buffered saline (PBS), and a final concentration of 1 μg/ml of each echinocandin in CSM medium was added to different wells. Fluorescence intensity (excitation at 485 nm and emission at 530 nm) was measured for 1 h in a microplate reader (Tecan Infinite M200 Pro). Mycelia stained with DCFDA without any drug were used as a control.

### RNA-seq.

RG101 conidia were grown for 16 h in YPD broth in the absence and presence of 1 μg/ml CAS at 37°C. Mycelia were washed twice with PBS. RNA was isolated using a Qiagen RNeasy plant minikit (catalog no. 74904). RNA-seq analysis was performed by LC Sciences, Houston, TX. StringTie was used to estimate expression levels for mRNAs by calculating fragments per kilobase per million (FPKM) as follows: FPKM = total number of exon fragments/number of mapped reads (millions) × exon length (in kilobases) (52). The differentially expressed mRNAs were selected with log2 (fold change) values of more than 1 or less than −1 and with a parametric F-test comparing nested linear models (*P* value, <0.05) by the use of R package Ballgown (53).

## References

[1] Brown GD, Denning DW, Gow NA, Levitz SM, Netea MG, White TC 2012 Hidden killers: human fungal infections. Sci Transl Med 4:165rv13. doi:10.1126/scitranslmed.3004404. 23253612

[2] Diekema D, Arbefeville S, Boyken L, Kroeger J, Pfaller M 2012 The changing epidemiology of healthcare-associated candidemia over three decades. Diagn Microbiol Infect Dis 73:45–48. doi:10.1016/j.diagmicrobio.2012.02.001. 22578938

[3] Jenks JD, Hoenigl M 2018 Treatment of aspergillosis. J Fungi (Basel) 4:98. doi:10.3390/jof4030098. PMC6162797 30126229

[4] Perlin DS 2011 Current perspectives on echinocandin class drugs. Future Microbiol 6:441–457. doi:10.2217/fmb.11.19. 21526945 PMC3913534

[5] Perlin DS 2015 Echinocandin resistance in Candida. Clin Infect Dis 61(Suppl 6):S612–S617. doi:10.1093/cid/civ791. 26567278 PMC4643482

[6] Jimenez-Ortigosa C, Moore C, Denning DW, Perlin DS 2017 Emergence of echinocandin resistance due to a point mutation in the fks1 gene of Aspergillus fumigatus in a patient with chronic pulmonary aspergillosis. Antimicrob Agents Chemother 61:e01277-17. doi:10.1128/AAC.01277-17. 28923871 PMC5700295

[7] Rocha EM, Garcia-Effron G, Park S, Perlin DS 2007 A Ser678Pro substitution in Fks1p confers resistance to echinocandin drugs in Aspergillus fumigatus. Antimicrob Agents Chemother 51:4174–4176. doi:10.1128/AAC.00917-07. 17724146 PMC2151465

[8] Satish S, Jimenez-Ortigosa C, Perlin DS 2018 Understanding the mechanistic basis of echinocandin resistance in *Aspergillus fumigatus*. Abstr Adv Aspergillosis, Lisbon, Portugal, 1–3 February 2018.

[9] Arendrup MC, Garcia-Effron G, Buzina W, Mortensen KL, Reiter N, Lundin C, Jensen HE, Lass-Flörl C, Perlin DS, Bruun B 2009 Breakthrough Aspergillus fumigatus and Candida albicans double infection during caspofungin treatment: laboratory characteristics and implication for susceptibility testing. Antimicrob Agents Chemother 53:1185–1193. doi:10.1128/AAC.01292-08. 19104024 PMC2650576

[10] Andaluz E, Guillen A, Larriba G 1986 Preliminary evidence for a glucan acceptor in the yeast Candida albicans. Biochem J 240:495–502. doi:10.1042/bj2400495. 2949741 PMC1147443

[11] Orlean PA 1982 (1,3)-Beta-d-glucan synthase from budding and filamentous cultures of the dimorphic fungus Candida albicans. Eur J Biochem 127:397–403. doi:10.1111/j.1432-1033.1982.tb06885.x. 6216107

[12] Ishiguro J, Saitou A, Duran A, Ribas JC 1997 cps1+, a Schizosaccharomyces pombe gene homolog of Saccharomyces cerevisiae FKS genes whose mutation confers hypersensitivity to cyclosporin A and papulacandin B. J Bacteriol 179:7653–7662. doi:10.1128/jb.179.24.7653-7662.1997. 9401022 PMC179726

[13] Kelly R, Register E, Hsu MJ, Kurtz M, Nielsen J 1996 Isolation of a gene involved in 1,3-beta-glucan synthesis in Aspergillus nidulans and purification of the corresponding protein. J Bacteriol 178:4381–4391. doi:10.1128/jb.178.15.4381-4391.1996. 8755864 PMC178203

[14] Mazur P, Morin N, Baginsky W, el-Sherbeini M, Clemas JA, Nielsen JB, Foor F 1995 Differential expression and function of two homologous subunits of yeast 1,3-beta-d-glucan synthase. Mol Cell Biol 15:5671–5681. doi:10.1128/MCB.15.10.5671. 7565718 PMC230817

[15] Mio T, Adachi-Shimizu M, Tachibana Y, Tabuchi H, Inoue SB, Yabe T, Yamada-Okabe T, Arisawa M, Watanabe T, Yamada-Okabe H 1997 Cloning of the Candida albicans homolog of Saccharomyces cerevisiae GSC1/FKS1 and its involvement in beta-1,3-glucan synthesis. J Bacteriol 179:4096–4105. doi:10.1128/jb.179.13.4096-4105.1997. 9209021 PMC179227

[16] Thompson JR, Douglas CM, Li W, Jue CK, Pramanik B, Yuan X, Rude TH, Toffaletti DL, Perfect JR, Kurtz M 1999 A glucan synthase FKS1 homolog in cryptococcus neoformans is single copy and encodes an essential function. J Bacteriol 181:444–453. 9882657 10.1128/jb.181.2.444-453.1999 PMC93397

[17] Johnson ME, Edlind TD 2012 Topological and mutational analysis of Saccharomyces cerevisiae Fks1. Eukaryot Cell 11:952–960. doi:10.1128/EC.00082-12. 22581527 PMC3416503

[18] Healey KR, Katiyar SK, Raj S, Edlind TD 2012 CRS-MIS in Candida glabrata: sphingolipids modulate echinocandin-Fks interaction. Mol Microbiol 86:303–313. doi:10.1111/j.1365-2958.2012.08194.x. 22909030 PMC3472958

[19] Gardiner RE, Souteropoulos P, Park S, Perlin DS 2005 Characterization of Aspergillus fumigatus mutants with reduced susceptibility to caspofungin. Med Mycol 43(Suppl 1):S299–S305. 16110824 10.1080/13693780400029023

[20] Walker LA, Gow NA, Munro CA 2013 Elevated chitin content reduces the susceptibility of Candida species to caspofungin. Antimicrob Agents Chemother 57:146–154. doi:10.1128/AAC.01486-12. 23089748 PMC3535899

[21] Park S, Kelly R, Kahn JN, Robles J, Hsu MJ, Register E, Li W, Vyas V, Fan H, Abruzzo G, Flattery A, Gill C, Chrebet G, Parent SA, Kurtz M, Teppler H, Douglas CM, Perlin DS 2005 Specific substitutions in the echinocandin target Fks1p account for reduced susceptibility of rare laboratory and clinical Candida sp. isolates. Antimicrob Agents Chemother 49:3264–3273. doi:10.1128/AAC.49.8.3264-3273.2005. 16048935 PMC1196231

[22] Meis JF, Chowdhary A, Rhodes JL, Fisher MC, Verweij PE 5 12 2016, posting date. Clinical implications of globally emerging azole resistance in Aspergillus fumigatus. Philos Trans R Soc Lond B Biol Sci doi:10.1098/rstb.2015.0460. PMC5095539 28080986

[23] Johnson MD, Perfect JR 2003 Caspofungin: first approved agent in a new class of antifungals. Expert Opin Pharmacother 4:807–823. doi:10.1517/14656566.4.5.807. 12740003

[24] Douglas CM, Marrinan JA, Li W, Kurtz MB 1994 A Saccharomyces cerevisiae mutant with echinocandin-resistant 1,3-beta-d-glucan synthase. J Bacteriol 176:5686–5696. doi:10.1128/jb.176.18.5686-5696.1994. 8083161 PMC196772

[25] Garcia-Effron G, Lee S, Park S, Cleary JD, Perlin DS 2009 Effect of Candida glabrata FKS1 and FKS2 mutations on echinocandin sensitivity and kinetics of 1,3-beta-d-glucan synthase: implication for the existing susceptibility breakpoint. Antimicrob Agents Chemother 53:3690–3699. doi:10.1128/AAC.00443-09. 19546367 PMC2737881

[26] Garcia-Effron G, Park S, Perlin DS 2009 Correlating echinocandin MIC and kinetic inhibition of fks1 mutant glucan synthases for Candida albicans: implications for interpretive breakpoints. Antimicrob Agents Chemother 53:112–122. doi:10.1128/AAC.01162-08. 18955538 PMC2612148

[27] Pfaller MA, Boyken L, Hollis RJ, Kroeger J, Messer SA, Tendolkar S, Diekema DJ 2008 In vitro susceptibility of invasive isolates of Candida spp. to anidulafungin, caspofungin, and micafungin: six years of global surveillance. J Clin Microbiol 46:150–156. doi:10.1128/JCM.01901-07. 18032613 PMC2224271

[28] Altwasser R, Baldin C, Weber J, Guthke R, Kniemeyer O, Brakhage AA, Linde J, Valiante V 2015 Network modeling reveals cross talk of MAP kinases during adaptation to caspofungin stress in Aspergillus fumigatus. PLoS One 10:e0136932. doi:10.1371/journal.pone.0136932. 26356475 PMC4565559

[29] Dunkel N, Liu TT, Barker KS, Homayouni R, Morschhauser J, Rogers PD 2008 A gain-of-function mutation in the transcription factor Upc2p causes upregulation of ergosterol biosynthesis genes and increased fluconazole resistance in a clinical Candida albicans isolate. Eukaryot Cell 7:1180–1190. doi:10.1128/EC.00103-08. 18487346 PMC2446669

[30] Loiko V, Wagener J 24 1 2017, posting date. The paradoxical effect of echinocandins in Aspergillus fumigatus relies on recovery of the beta-1,3-glucan synthase Fks1. Antimicrob Agents Chemother doi:10.1128/AAC.01690-16. PMC5278722 27872079

[31] Healey KR, Katiyar SK, Castanheira M, Pfaller MA, Edlind TD 2011 Candida glabrata mutants demonstrating paradoxical reduced caspofungin susceptibility but increased micafungin susceptibility. Antimicrob Agents Chemother 55:3947–3949. doi:10.1128/AAC.00044-11. 21628537 PMC3147630

[32] Gao J, Wang H, Li Z, Wong AH-H, Wang Y-Z, Guo Y, Lin X, Zeng G, Liu H, Wang Y, Wang J 2018 Candida albicans gains azole resistance by altering sphingolipid composition. Nat Commun 9:4495. doi:10.1038/s41467-018-06944-1. 30374049 PMC6206040

[33] Vanden Bossche H, Warnock DW, Dupont B, Kerridge D, Sen Gupta S, Improvisi L, Marichal P, Odds FC, Provost F, Ronin O 1994 Mechanisms and clinical impact of antifungal drug resistance. J Med Vet Mycol 32(Suppl 1):189–202. doi:10.1080/02681219480000821. 7722785

[34] Brajtburg J, Powderly WG, Kobayashi GS, Medoff G 1990 Amphotericin B: current understanding of mechanisms of action. Antimicrob Agents Chemother 34:183–188. doi:10.1128/AAC.34.2.183. 2183713 PMC171553

[35] Lamy-Freund MT, Ferreira VF, Schreier S 1985 Mechanism of inactivation of the polyene antibiotic amphotericin B. Evidence for radical formation in the process of autooxidation. J Antibiot (Tokyo) 38:753–757. doi:10.7164/antibiotics.38.753. 2991182

[36] Moore CB, Sayers N, Mosquera J, Slaven J, Denning DW 2000 Antifungal drug resistance in Aspergillus. J Infect 41:203–220. doi:10.1053/jinf.2000.0747. 11120607

[37] Shekhova E, Kniemeyer O, Brakhage AA 2017 Induction of mitochondrial reactive oxygen species production by itraconazole, terbinafine, and amphotericin B as a mode of action against Aspergillus fumigatus. Antimicrob Agents Chemother 61:e00978-17. doi:10.1128/AAC.00978-17. 28848005 PMC5655112

[38] Stevens DA, Espiritu M, Parmar R 2004 Paradoxical effect of caspofungin: reduced activity against Candida albicans at high drug concentrations. Antimicrob Agents Chemother 48:3407–3411. doi:10.1128/AAC.48.9.3407-3411.2004. 15328104 PMC514730

[39] Wagener J, Loiko V 28 12 2017, posting date. Recent insights into the paradoxical effect of echinocandins. J Fungi (Basel) doi:10.3390/jof4010005. PMC5872308 29371498

[40] Fortwendel JR, Juvvadi PR, Perfect BZ, Rogg LE, Perfect JR, Steinbach WJ 2010 Transcriptional regulation of chitin synthases by calcineurin controls paradoxical growth of Aspergillus fumigatus in response to caspofungin. Antimicrob Agents Chemother 54:1555–1563. doi:10.1128/AAC.00854-09. 20124000 PMC2849361

[41] Stevens DA, Ichinomiya M, Koshi Y, Horiuchi H 2006 Escape of Candida from caspofungin inhibition at concentrations above the MIC (paradoxical effect) accomplished by increased cell wall chitin; evidence for beta-1,6-glucan synthesis inhibition by caspofungin. Antimicrob Agents Chemother 50:3160–3161. doi:10.1128/AAC.00563-06. 16940118 PMC1563524

[42] Shingu-Vazquez M, Traven A 2011 Mitochondria and fungal pathogenesis: drug tolerance, virulence, and potential for antifungal therapy. Eukaryot Cell 10:1376–1383. doi:10.1128/EC.05184-11. 21926328 PMC3209048

[43] Dagley MJ, Gentle IE, Beilharz TH, Pettolino FA, Djordjevic JT, Lo TL, Uwamahoro N, Rupasinghe T, Tull DL, McConville M, Beaurepaire C, Nantel A, Lithgow T, Mitchell AP, Traven A 2011 Cell wall integrity is linked to mitochondria and phospholipid homeostasis in Candida albicans through the activity of the post-transcriptional regulator Ccr4-Pop2. Mol Microbiol 79:968–989. doi:10.1111/j.1365-2958.2010.07503.x. 21299651

[44] Kornmann B, Currie E, Collins SR, Schuldiner M, Nunnari J, Weissman JS, Walter P 2009 An ER-mitochondria tethering complex revealed by a synthetic biology screen. Science 325:477–481. doi:10.1126/science.1175088. 19556461 PMC2933203

[45] Osman C, Haag M, Potting C, Rodenfels J, Dip PV, Wieland FT, Brugger B, Westermann B, Langer T 2009 The genetic interactome of prohibitins: coordinated control of cardiolipin and phosphatidylethanolamine by conserved regulators in mitochondria. J Cell Biol 184:583–596. doi:10.1083/jcb.200810189. 19221197 PMC2654118

[46] Tamura Y, Endo T, Iijima M, Sesaki H 2009 Ups1p and Ups2p antagonistically regulate cardiolipin metabolism in mitochondria. J Cell Biol 185:1029–1045. doi:10.1083/jcb.200812018. 19506038 PMC2711612

[47] Niles BJ, Joslin AC, Fresques T, Powers T 2014 TOR complex 2-Ypk1 signaling maintains sphingolipid homeostasis by sensing and regulating ROS accumulation. Cell Rep 6:541–552. doi:10.1016/j.celrep.2013.12.040. 24462291 PMC3985744

[48] CLSI. 2008 Reference method for broth dilution antifungal susceptibility testing of filamentous fungi; approved standard. Document M38-A2, vol 28, no. 16 National Committee for Clinical Laboratory Standards, Wayne, Pa.

[49] Nishiyama Y, Hasumi Y, Ueda K, Uchida K, Yamaguchi H 2005 Effects of micafungin on the morphology of Aspergillus fumigatus. J Electron Microsc (Tokyo) 54:67–77. doi:10.1093/jmicro/dfh100. 15695488

[50] Bielawski J, Pierce JS, Snider J, Rembiesa B, Szulc ZM, Bielawska A 2010 Sphingolipid analysis by high performance liquid chromatography-tandem mass spectrometry (HPLC-MS/MS). Adv Exp Med Biol 688:46–59. doi:10.1007/978-1-4419-6741-1_3. 20919645

[51] Blatzer M, Jukic E, Posch W, Schöpf B, Binder U, Steger M, Blum G, Hackl H, Gnaiger E, Lass-Flörl C, Wilflingseder D 2015 Amphotericin B resistance in Aspergillus terreus is overpowered by coapplication of pro-oxidants. Antioxid Redox Signal 23:1424–1438. doi:10.1089/ars.2014.6220. 26054424

[52] Pertea M, Pertea GM, Antonescu CM, Chang TC, Mendell JT, Salzberg SL 2015 StringTie enables improved reconstruction of a transcriptome from RNA-seq reads. Nat Biotechnol 33:290–295. doi:10.1038/nbt.3122. 25690850 PMC4643835

[53] Frazee AC, Pertea G, Jaffe AE, Langmead B, Salzberg SL, Leek JT 2015 Ballgown bridges the gap between transcriptome assembly and expression analysis. Nat Biotechnol 33:243–246. doi:10.1038/nbt.3172. 25748911 PMC4792117

