# Supplementary material for: Stress-Induced Changes in the Lipid Microenvironment of β-(1,3)-d-Glucan Synthase Cause Clinically Important Echinocandin Resistance in Aspergillus fumigatus
Source: mBio. 2019 Jun 4;10(3):e00779-19. doi: 10.1128/mBio.00779-19 (PMC6550521; doi:10.1128/mBio.00779-19)
Supplement: FIG S1 [file mBio.00779-19-sf001.docx]

**FIGURE S1**

**FIG S1: *fks1* expression levels in RG101 in uninduced and CAS-induced conditions.** RG101 conidia were grown for 16 h in YPD in the absence and presence of CAS (1 and 4 µg/mL) and expression levels of *fks1* were compared using RT-PCR. No significant difference in *fks1* expression levels was seen between uninduced and CAS-induced conditions (p > 0.05).
