# Supplementary material for: Stress-Induced Changes in the Lipid Microenvironment of β-(1,3)-d-Glucan Synthase Cause Clinically Important Echinocandin Resistance in Aspergillus fumigatus
Source: mBio. 2019 Jun 4;10(3):e00779-19. doi: 10.1128/mBio.00779-19 (PMC6550521; doi:10.1128/mBio.00779-19)
Supplement: FIG S2 [file mBio.00779-19-sf002.docx]

**FIGURE S2**


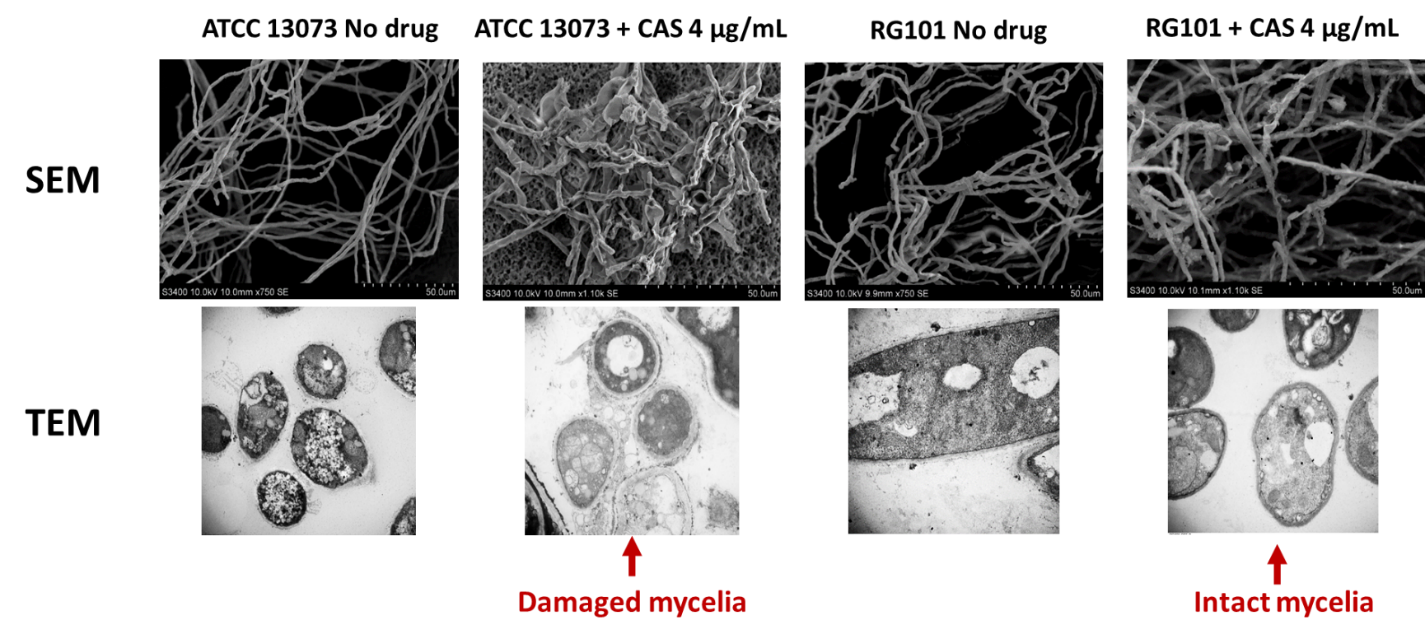


**FIG S2: Cell wall ultrastructure of RG101 in the presence of CAS.** Conidia of ATCC 13073 and RG101 strains were grown in liquid YPD in the presence of CAS (4 µg/mL) for 16 h and processed for SEM and TEM imaging. Electron microscopy images showed no morphological defects in RG101 grown in the presence of CAS, whereas the CAS-sensitive parental strain ATCC 13073 showed irregular mycelia and leaking cellular contents indicating damaged cell wall.
