# Supplementary material for: Stress-Induced Changes in the Lipid Microenvironment of β-(1,3)-d-Glucan Synthase Cause Clinically Important Echinocandin Resistance in Aspergillus fumigatus
Source: mBio. 2019 Jun 4;10(3):e00779-19. doi: 10.1128/mBio.00779-19 (PMC6550521; doi:10.1128/mBio.00779-19)
Supplement: FIG S3 [file mBio.00779-19-sf003.docx]

**
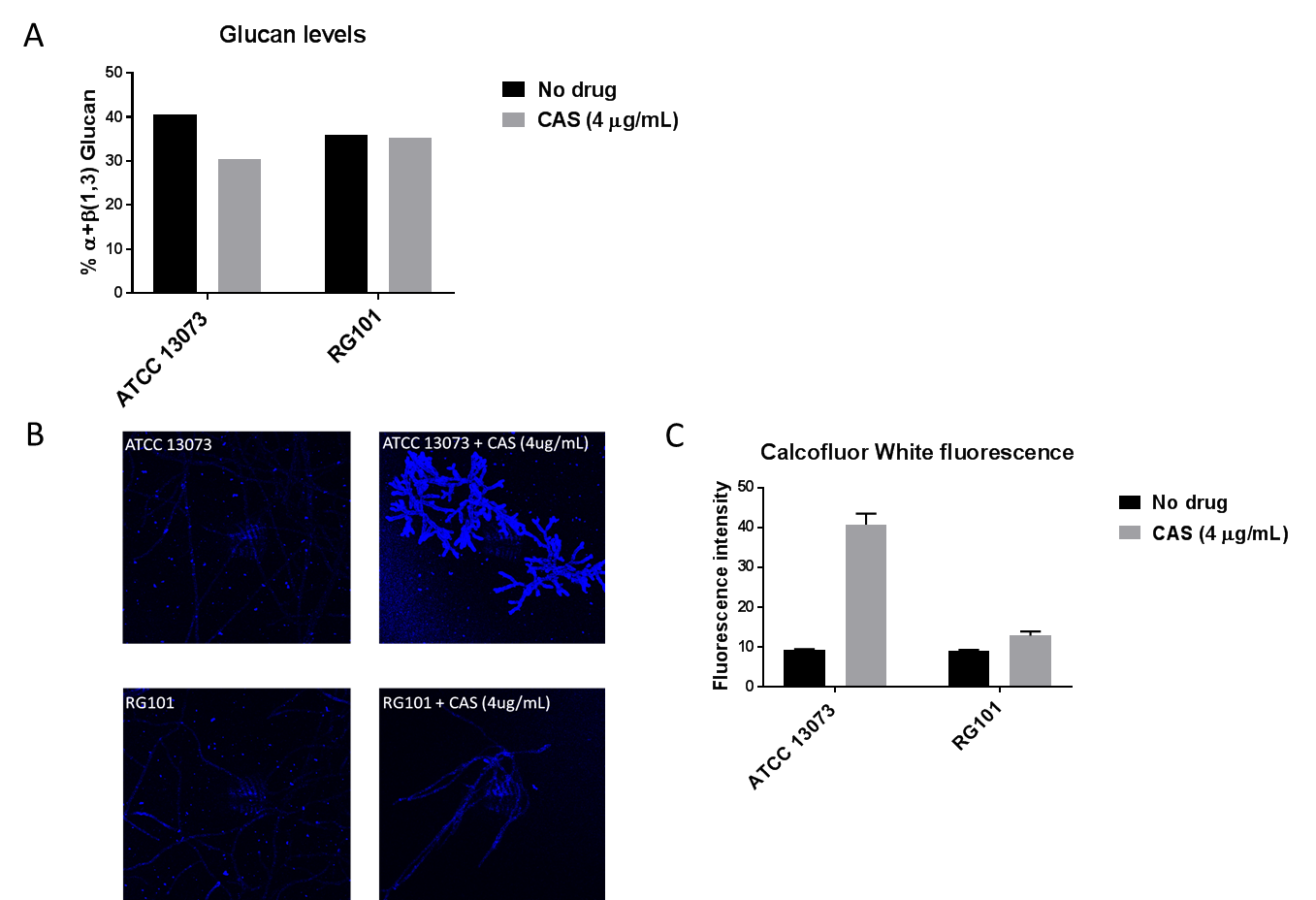
FIGURE S3**

**FIG S3: Evaluation of glucan and chitin contents of RG101 cell wall:** (A) Biochemical analysis of cell wall glucan levels using Mass Spectroscopy showing no reduction in glucan content in RG101 treated with CAS. (B, C) Fluorescence study using calcofluor white showing no increase in chitin levels in RG101 treated with CAS (4 µg/mL). In summary, no cell wall associated changes were detected in RG101 treated with CAS, indicating complete resistance to the drug.
