# Supplementary material for: Stress-Induced Changes in the Lipid Microenvironment of β-(1,3)-d-Glucan Synthase Cause Clinically Important Echinocandin Resistance in Aspergillus fumigatus
Source: mBio. 2019 Jun 4;10(3):e00779-19. doi: 10.1128/mBio.00779-19 (PMC6550521; doi:10.1128/mBio.00779-19)
Supplement: FIG S4 [file mBio.00779-19-sf004.docx]

**
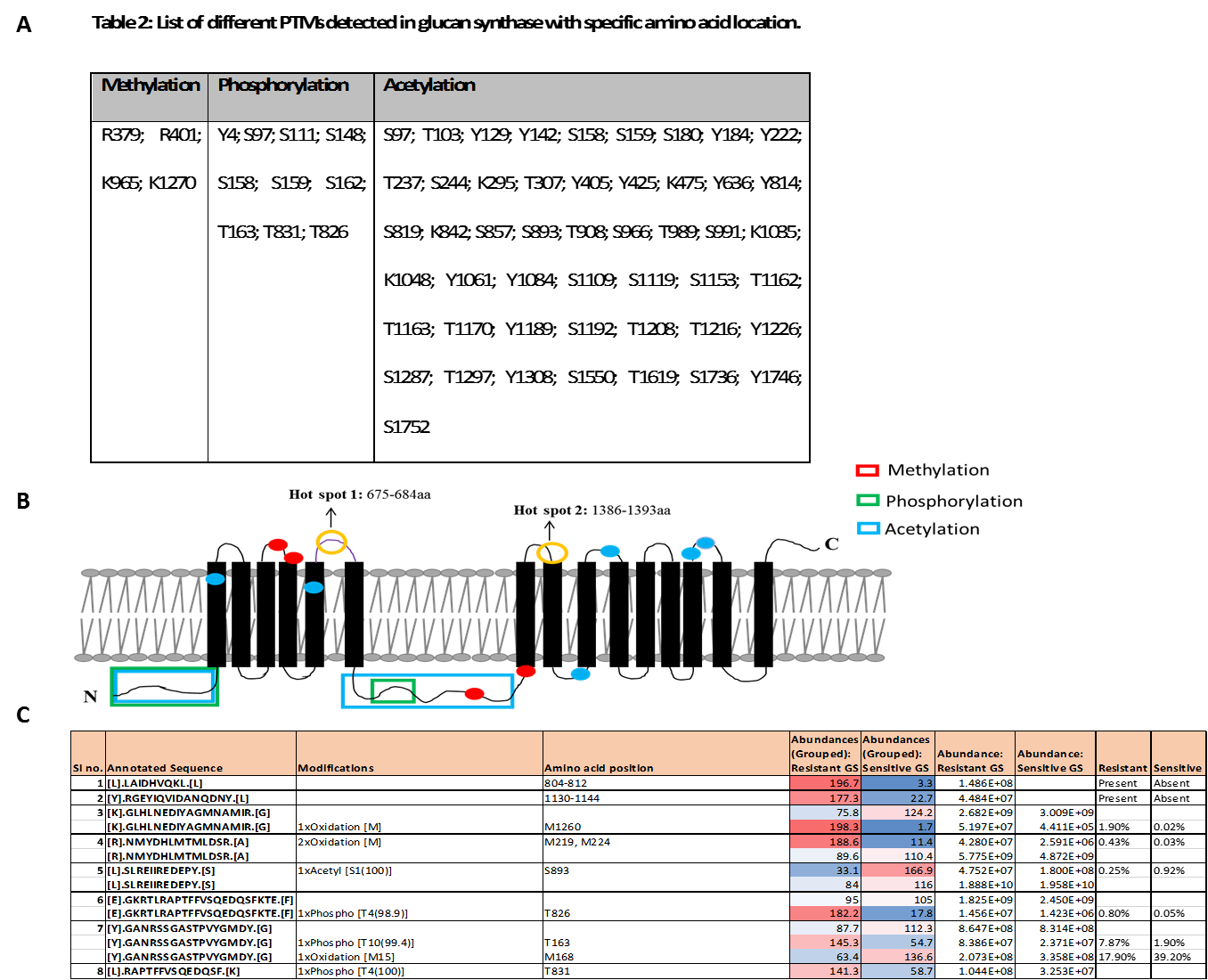
FIGURE S4**

**FIG S4: PTMs identified in Glucan Synthase:** Nano LC-MS of glucan synthase of RG101 after enzyme digestion covered 71.5% of the protein and detected several PTMs. (A) A list of different PTMs detected with specific amino acid location. (B) A diagrammatic representation of glucan synthase 16 transmembrane domains and the overall distribution of PTMs detected. Most of the PTMs were studded in the cytosolic segments of the protein. No PTMs were detected in the two hot-spot regions of the enzyme. (C) List of 8 peptides showing difference in PTMs between CAS induced and uninduced enzymes. However, very low fraction of the population of the enzyme was modified, which did not account for the observed phenotype in RG101.
