# Supplementary material for: Stress-Induced Changes in the Lipid Microenvironment of β-(1,3)-d-Glucan Synthase Cause Clinically Important Echinocandin Resistance in Aspergillus fumigatus
Source: mBio. 2019 Jun 4;10(3):e00779-19. doi: 10.1128/mBio.00779-19 (PMC6550521; doi:10.1128/mBio.00779-19)
Supplement: FIG S5 [file mBio.00779-19-sf005.docx]

**FIGURE S5**

**
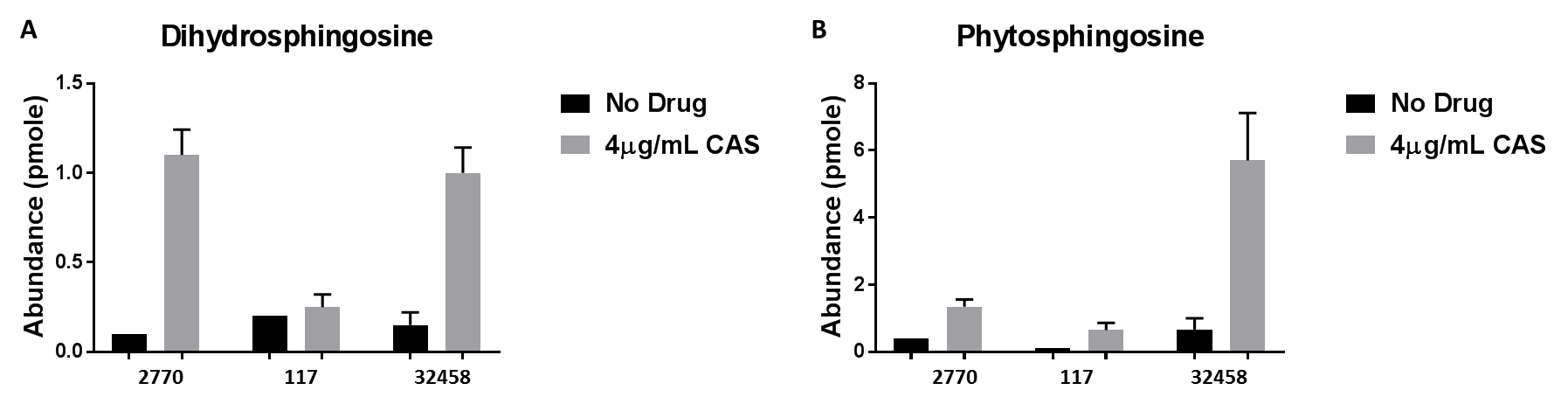
**

**FIG S5: Abundance levels of DhSph and PhSph lipid species in clinical isolates:** Glucan synthase of three clinical isolates listed in Table 1- 2770, 117 and 32458 - were tested for abundance levels of DhSph and PhSph under uninduced and induced conditions. Consistent with data from RG101, these clinical isolates also showed higher levels of DhSph (A) and PhSph (B) in CAS-induced enzymes compared to uninduced enzyme preparation. This indicated that CAS-induces lipid modifications in clinical isolates, as observed in RG101.
