# Supplementary material for: Stress-Induced Changes in the Lipid Microenvironment of β-(1,3)-d-Glucan Synthase Cause Clinically Important Echinocandin Resistance in Aspergillus fumigatus
Source: mBio. 2019 Jun 4;10(3):e00779-19. doi: 10.1128/mBio.00779-19 (PMC6550521; doi:10.1128/mBio.00779-19)
Supplement: TABLE S1 [file mBio.00779-19-st001.docx]

**TABLE S1**

|  | **Lipid type** | **RG101 gel- No drug** | **RG101 + CAS (1 µg/mL) gel** |  |  | **Lipid type** | **RG101 gel- No drug** | **RG101 + CAS (1 µg/mL) gel** |
| --- | --- | --- | --- | --- | --- | --- | --- | --- |
| 1 | C14-Cer | 0.0 | 0.002 |  |  |  |  |  |
| 2 | C16-Cer | 0.0 | 0.003 |  | 24 | dhC26-Cer | 0.1 | 0.012 |
| 3 | C18-Cer | 0.4 | 0.544 |  | 25 | dhC26:1-Cer | 0.0 | 0.002 |
| 4 | C18:1-Cer | 0.3 | 0.198 |  | **26** | **dhSph** | **0.1** | **0.358** |
| 5 | C20-Cer | 0.1 | 0.022 |  | 27 | dhSph-1P | 0.0 | 0.000 |
| 6 | C20:1-Cer | 0.0 | 0.007 |  | 28 | Sph | 0.2 | 0.111 |
| 7 | C20:4-Cer | 0.0 | 0.000 |  | 29 | Sph-1P | 0.0 | 0.001 |
| 8 | C22-Cer | 0.1 | 0.017 |  | **30** | **Phyto-Sph** | **5.6** | **57.448** |
| 9 | C22:1-Cer | 0.0 | 0.006 |  | 31 | Phyto-Sph-1P | 0.0 | 0.017 |
| 10 | C24-Cer | 0.2 | 0.039 |  | 32 | PhytoC14-Cer | 0.0 | 0.005 |
| 11 | C24:1-Cer | 0.2 | 0.047 |  | 33 | PhytoC16-Cer | 0.0 | 0.010 |
| 12 | C26-Cer | 0.0 | 0.003 |  | 34 | PhytoC18-Cer | 0.1 | 0.079 |
| 13 | C26:1-Cer | 0.0 | 0.001 |  | 35 | PhytoC18:1-Cer | 0.0 | 0.021 |
| 14 | dhC14-Cer | 0.0 | 0.001 |  | 36 | PhytoC20-Cer | 0.0 | 0.018 |
| 15 | dhC16-Cer | 0.0 | 0.002 |  | 37 | PhytoC20:1-Cer | 0.0 | 0.002 |
| 16 | dhC18-Cer | 0.6 | 0.907 |  | 38 | PhytoC22-Cer | 0.1 | 0.610 |
| 17 | dhC18:1-Cer | 0.0 | 0.004 |  | 39 | PhytoC22:1-Cer | 0.0 | 0.008 |
| 18 | dhC20-Cer | 0.3 | 0.310 |  | **40** | **PhytoC24-Cer** | **4.9** | **13.312** |
| 19 | dhC20:1-Cer | 0.1 | 0.134 |  | 41 | PhytoC24:1-Cer | 0.0 | 0.094 |
| 20 | dhC22-Cer | 0.0 | 0.006 |  | 42 | PhytoC26-Cer | 0.1 | 0.157 |
| 21 | dhC22:1-Cer | 0.0 | 0.014 |  | 43 | PhytoC26:1-Cer | 0.0 | 0.009 |
| 22 | dhC24-Cer | 0.0 | 0.005 |  | 44 | PhytoC28-Cer | 0.0 | 0.001 |
| 23 | dhC24:1-Cer | 0.0 | 0.014 |  | 45 | PhytoC28:1-Cer | 0.0 | 0.000 |

**Table S1: Relative abundance of different lipid species in the microenvironment of glucan synthase.** Three lipid sub-types (in red) - dihydrospingosine (DhSph), phytosphingosine (PhSph) and phytoceramide (C24) - were present 3-, 10-, and 3-fold higher, respectively, in CAS-induced glucan synthase of RG101 compared to the uninduced preparation. DhSph and PhSph were used for further analysis.
