# Supplementary material for: Stress-Induced Changes in the Lipid Microenvironment of β-(1,3)-d-Glucan Synthase Cause Clinically Important Echinocandin Resistance in Aspergillus fumigatus
Source: mBio. 2019 Jun 4;10(3):e00779-19. doi: 10.1128/mBio.00779-19 (PMC6550521; doi:10.1128/mBio.00779-19)
Supplement: FIG S6 [file mBio.00779-19-sf006.docx]

**FIGURE S6**


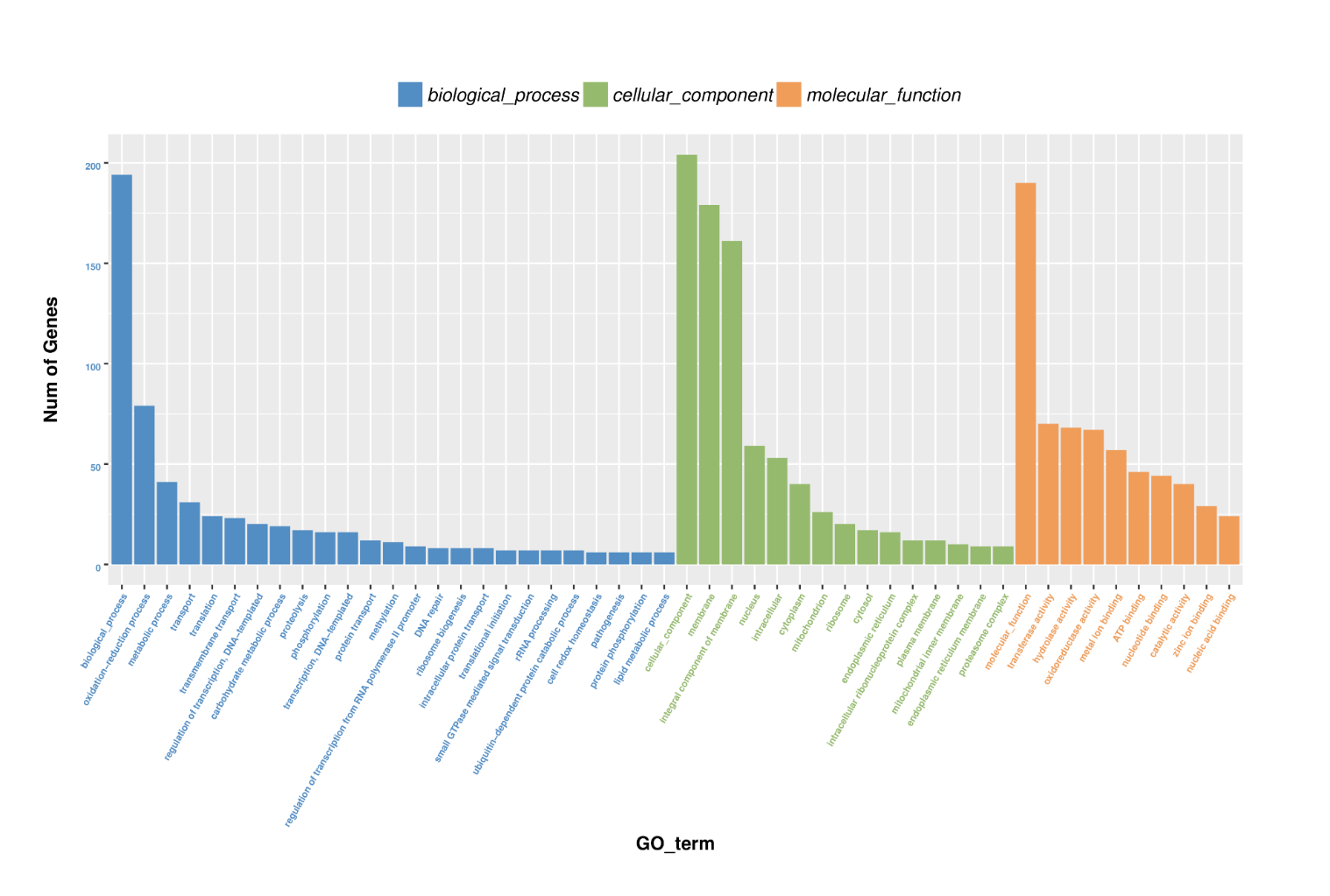


**FIG S6: Differentially expressed genes with their gene ontology between CAS induced and uninduced conditions in RG101.** RG101 was grown in the presence and absence of CAS (1 µg/mL) for 16 hours, cells were isolated and processed for RNA seq. Genes involved in oxidation-reduction pathway were highly differentially expressed, consistent with the induction of ROS by CAS.
