# Supplementary material for: Stress-Induced Changes in the Lipid Microenvironment of β-(1,3)-d-Glucan Synthase Cause Clinically Important Echinocandin Resistance in Aspergillus fumigatus
Source: mBio. 2019 Jun 4;10(3):e00779-19. doi: 10.1128/mBio.00779-19 (PMC6550521; doi:10.1128/mBio.00779-19)
Supplement: TEXT S1 [file mBio.00779-19-s0001.docx]

**TEXT S1**

**TEXT S1: Materials and Methods**

***In vivo* fungal burden analysis**

All animal experiments performed were approved by the Rutgers University Institutional Animal Care and Use Committee. Thirty female DBA/2 mice [The Jackson Laboratory, Bar Harbor, ME] were used in the experiment. The mice were housed in cages equipped with HEPA filters and were allowed free access to food and water. To establish neutropenia, mice were immunosuppressed with cyclophosphamide on days -4 (150 mg/kg), -1 and +2 (100 mg/kg) intraperitoneally (ip) and hydrocortisone on day -1 (200 mg/kg) via subcutaneous injection (sc). The animals were given prophylaxis sc ceftazidime (50 mg/kg) on all days to prevent secondary bacterial infections. A. fumigatus RG101 was streaked out from a frozen glycerol stock and cultured on Potato dextrose agar (PDA) slant for 2 days. The spores were harvested in phosphate-buffered saline (PBS) with 0.05% (v/v) Tween 20 and counted in a hemocytometer. The mice were briefly anesthetized IP Ketamine (65 mg/kg)/Xylazine (5 mg/kg) and infected with 1×10^6^ spores of RG101 in 25 µL volume via intratracheal instillation. The 30 RG101 infected mice were randomized into following treatment arms for IP treatment: vehicle, CAS (5 mg/kg), and MFG (5 mg/kg). The first treatment was given at 3 h post infection (PI) and continued daily treatments were administered. On day 5 PI, mice were humanely euthanized via CO_2_ narcosis. Lungs were excised and homogenized in 1 ml of sterile PBS using a 2 ml tube containing ceramic spheres by MP Biomedicals (116913500). Tissue was then homogenized in 20 s bursts in a mini bead beater (MP FastPrep-24) followed by cooling on ice. DNA was then isolated from an aliquot of the lung homogenate (100 μl) using the QIAamp DNA Mini Kit by Qiagen (51304) and analyzed by real-time quantitative PCR (qPCR) using primers specific for A. fumigatus 18S rRNA genes (Wiederhold et al 2004).

**Protein analysis by LC-MS/MS**

For mass spectrometry analysis, an in-gel digestion of glucan synthase using trypsin-LysC, chymotrypsin and GluC (all Promega, Madison, WI) was performed according to the protocol (Shevchenko et al 2006). Peptides were then extracted with trifluoroacetic acid and increasing concentrations of acetonitrile (50-90%). Dried peptides were solubilized in 0.05% (v/v) trifluoroacetic acid in 2% (v/v) acetonitrile in water and used for LC-MS/MS analysis, which was carried out on an Ultimate 3000 nano-rapid-separation liquid chromatography (nano-RSLC) system coupled to a QExactive HF mass spectrometer (both from Thermo Fisher Scientific). Measurements were performed as previously described (Thurich et al 2018), except the following changes: gradient elution with eluents A (0.1% (v/v) formic acid in H_2_O) and B (0.1% (v/v) formic acid in acetonitrile/H_2_O (90%/10%, v/v) was as follows: 0-5 min at 4% B, 30 min at 7% B, 60 min at 10% B, 100 min at 15 % B, 140 min at 25% B, 180 min at 45% B, 200 min at 65% B, 210-215 min at 96% B, 215.1-240 min at 4% B. The AGC (automatic gain control) target for the precursor ion scan was set to 3e6 and the maximum injection time for both precursor and MS2 ions was set to 120 ms. Tandem mass spectra were searched against the UniProt database (2018/01/24; https://www.uniprot.org/proteomes/UP000002530) of *Neosartorya fumigata* (*Aspergillus fumigatus*) Af293, using Proteome Discoverer (PD) 2.2 (Thermo) and the algorithms of Mascot 2.4 Sequest HT (version of PD2.2) and MS Amanda 2.0. Two missed cleavages were allowed for the proteolytic digestion. The precursor mass tolerance was set to 10 ppm and the fragment mass tolerance was set to 0.02 Da. Dynamic modifications of amino acid residues were oxidation (M), phosphorylation (S, T, Y), acetylation (K, S, T, Y, protein N-term), methylation (D, E, K, R), and dimethylation (K, R). Static modification was carbamidomethylation (C). At least 2 peptides per protein and a strict false discovery rate (FDR) < 1% (peptide and protein level) were required for positive protein hits. The Percolator node of PD2.2 and a reverse decoy database was used for q-value validation of spectral matches. Only rank 1 proteins and peptides of the top scored proteins were counted. Label-free protein quantification was based on the Minora algorithm of PD2.2 using a signal-to-noise ratio >5. Normalization was performed by using the total peptide amount method. Imputation of missing quan values was applied by using the replicate based resampling method. The mass spectrometry proteomics data have been deposited to the ProteomeXchange Consortium via the PRIDE (Vizcaino et al 2016) partner repository with the dataset identifier PXD012438.

**Electron microscopy**

For SEM, mycelia of ATCC 13073 and RG101 were grown in the absence and presence of CAS (4 µg/mL) and were fixed with 2.5% (v/v) glutaraldehyde in 0.1 M cacodylate buffer (pH 7.4) at room temperature for 3 h. After being washed with 0.1 M cacodylate buffer, specimens were post-fixed for 1 h with 1% osmium tetroxide in 0.1 M cacodylate buffer (pH 7.4) at 4^°^C. Samples were dehydrated in graded ethanol, and then critical point dried in liquid carbon dioxide and sputter-coated with a thin layer of palladium–gold. Observations were carried out using Hitachi S-3400 N scanning electron microscope.

For TEM, cultures were fixed with 2.5% (v/v) glutaraldehyde solution as described above. They were then post-fixed for 1 h with 1% (w/v) osmium tetroxide in 0.1 M cacodylate buffer (pH 7.4) at 4^°^C. After three wash steps in buffer, specimens were dehydrated in a series of graded ethanol and embedded in Embed 812. Thin sections were stained with uranyl acetate followed by lead nitrate and examined in a Hitachi H-7500 electron microscope operated at 100 KV.

**REFERENCES**

Shevchenko A, Tomas H, Havlis J, Olsen JV, Mann M. 2006. In-gel digestion for mass spectrometric characterization of proteins and proteomes. *Nat Protoc* 1: 2856-60

Thurich J, Meichsner D, Furch ACU, Pfalz J, Kruger T, et al. 2018. Arabidopsis thaliana responds to colonisation of Piriformospora indica by secretion of symbiosis-specific proteins. *PLoS One* 13: e0209658

Vizcaino JA, Csordas A, Del-Toro N, Dianes JA, Griss J, et al. 2016. 2016 update of the PRIDE database and its related tools. *Nucleic Acids Res* 44: 11033

Wiederhold NP, Kontoyiannis DP, Chi J, Prince RA, Tam VH, Lewis RE. 2004. Pharmacodynamics of caspofungin in a murine model of invasive pulmonary aspergillosis: evidence of concentration-dependent activity. *J Infect Dis* 190: 1464-71
